# Brain organoid methodologies to explore mechanisms of disease in progressive multiple sclerosis

**DOI:** 10.3389/fncel.2024.1488691

**Published:** 2024-12-18

**Authors:** Madalena B. C. Simões-Abade, Marlene Patterer, Alexandra M. Nicaise, Stefano Pluchino

**Affiliations:** Department of Clinical Neurosciences and NIHR Biomedical Research Centre, University of Cambridge, Cambridge, United Kingdom

**Keywords:** progressive multiple sclerosis, smoldering inflammation, stem cells, disease modeling, neuroimmunology, brain organoids, precision medicine, regenerative neuroimmunology

## Abstract

Multiple sclerosis (MS), a debilitating autoimmune disorder targeting the central nervous system (CNS), is marked by relentless demyelination and inflammation. Clinically, it presents in three distinct forms: relapsing–remitting MS (RRMS), primary progressive MS (PPMS), and secondary progressive MS (SPMS). While disease-modifying therapies (DMTs) offer some relief to people with RRMS, treatment options for progressive MS (pMS) remain frustratingly inadequate. This gap highlights an urgent need for advanced disease modeling techniques to unravel the intricate pathology of pMS. Human induced pluripotent stem cell (iPSC) technologies and brain organoids are emerging as promising tools for disease modeling in both 2D and 3D *in vitro* environments. These innovative approaches enable the study of disease mechanisms that closely mimic human pathophysiology and offer new platforms for screening therapeutic compounds, surpassing the limitations of traditional animal models. However, deploying brain organoids in disease modeling presents challenges, especially in the context of non-monogenic disorders. This review delves into cutting-edge brain organoid techniques that hold the potential to revolutionize our understanding of pMS, offering a pathway to disentangle its underlying mechanisms and drive transformative discoveries.

## 1 Introduction

Multiple sclerosis (MS) is the most common autoimmune disorder affecting the central nervous system (CNS), characterized by both neuroinflammation and neurodegeneration (Compston and Coles, 2008). Despite genetic and environmental risk factors, including Epstein–Barr virus infection and lack of vitamin D, the etiology of MS remains unknown (Olsson et al., 2016). MS can be classified into three main forms: relapsing–remitting MS (RRMS), primary progressive MS (PPMS), and secondary progressive MS (SPMS). Typically, the course of MS starts with RRMS, usually diagnosed in younger ages (20s-30s), during which symptoms appear suddenly and are followed by a period of recovery. With age, this course usually evolves into SPMS marked by a progressive decline in neurological function. In contrast, approximately 10–15% of patients present with PPMS, where symptoms progressively worsen from onset without distinct relapses or recovery periods (Ghasemi et al., 2017).

**Figure 1:**
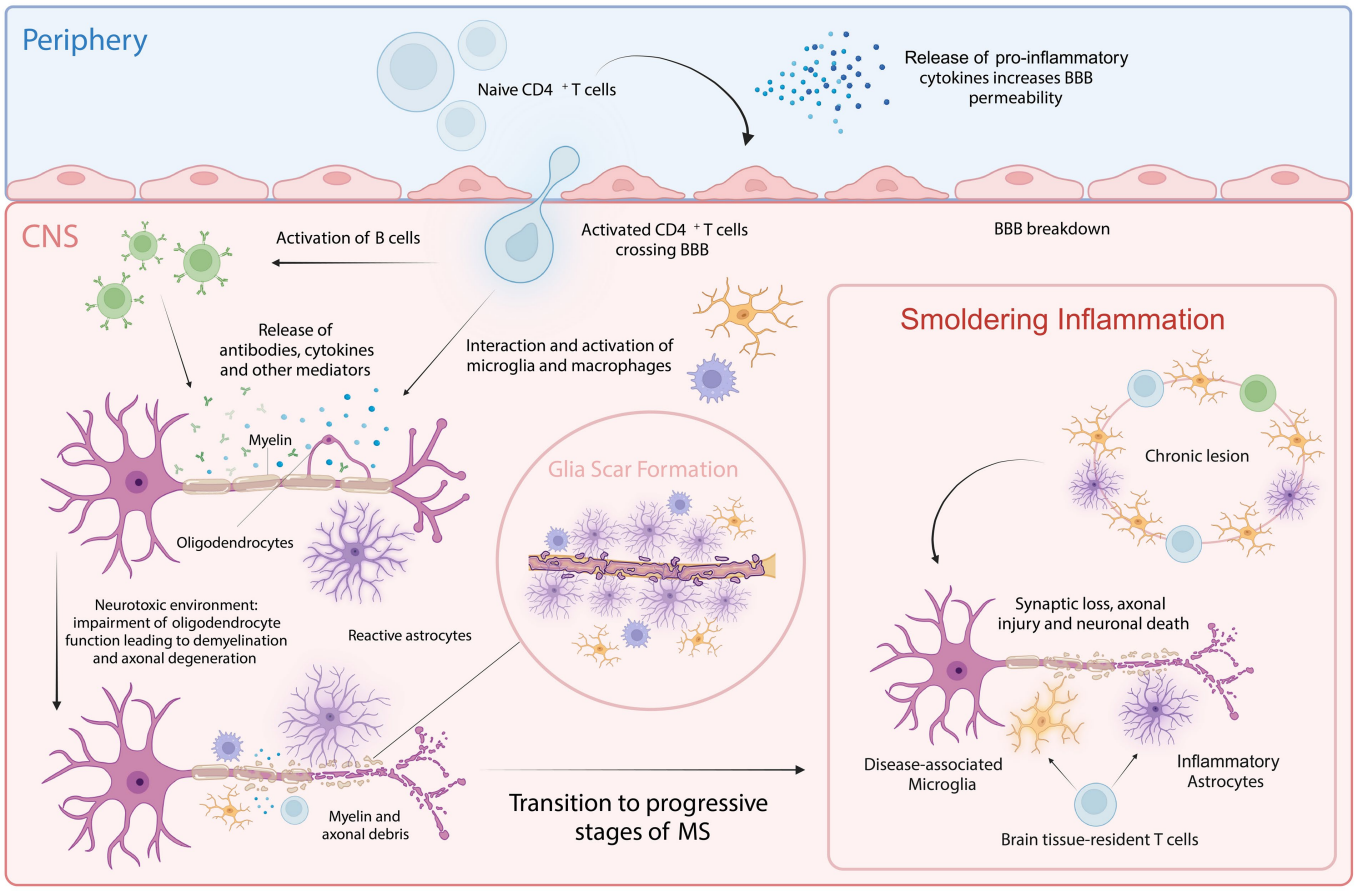
Cellular key players involved in MS pathology. In the periphery, upon activation of naive CD4^+^ T cells, pro-inflammatory cytokines are released that increase BBB permeability, allowing the autoreactive CD4^+^ T cells to cross into the CNS. Within the CNS, these T cells interact with and activate resident microglia, infiltrating macrophages and B cells. This interaction amplifies inflammatory response, leading to further recruitment of immune cells and the activation of astrocytes. This process is facilitated by both cytotoxic mechanisms and the release of autoantibodies and cytokines. Chronic inflammation and astrocyte reactivity contribute to glial scar formation, a hallmark of progressive MS. Smoldering inflammation persists within the CNS, marked by ongoing low-level immune activation and the gradual expansion of lesions, further driving neurodegeneration and characterizing progressive stages of MS. Created with BioRender.com.

Pathologically, MS is characterized by the disruption of the blood–brain barrier (BBB), allowing peripheral immune cells to infiltrate the CNS. These infiltrating immune cells target, and attack myelin sheaths produced by oligodendrocytes, leading to inflammation and demyelination. The release of pro-inflammatory cytokines, such as interleukins (ILs), interferons (IFNs), and tumor necrosis factors (TNFs), exacerbates this inflammation and damages both nerve fibers and oligodendrocytes, resulting in lesion formation (Ortiz et al., 2014; Weissert, 2017). As MS progresses, persistent inflammation activates astrocytes and microglia contributing to smoldering inflammation and neurodegeneration (Giovannoni et al., 2022). This sustained low-grade inflammation, driven by continuously activated microglia and astrocytes, leads to the release of pro-inflammatory cytokines and reactive oxygen species, creating a neurotoxic environment that disrupts neuronal homeostasis (Giovannoni et al., 2022). In the early stages of MS, remyelination can occur due to surviving oligodendrocytes and oligodendrocyte progenitor cells (OPCs). However, as the disease advances, the depletion of oligodendrocytes and impaired OPC function, compounded by persistent inflammation, result in remyelination failure (Goldschmidt et al., 2009; Klotz et al., 2023). This reduction in regenerative capacity further aggravates the neurotoxic condition of the CNS. Additionally, mitochondrial dysfunction, oxidative stress, and excitotoxicity contribute to synaptic loss, axonal injury, and eventually neuronal death (Lassmann et al., 2012; Figure 1). This ongoing neurodegenerative process underlies the progressive neurological decline observed in progressive MS (pMS; Woo et al., 2024). Despite significant advances in understanding MS, the precise mechanisms driving disease progression remain poorly understood. It is believed that aging plays a critical role in the transition from the inflammatory-dominant phase of RRMS to the neurodegenerative phase of SPMS, being a primary risk factor for the development of progressive MS forms, like other neurodegenerative diseases (Sanai et al., 2016; Musella et al., 2018; Hou et al., 2019).

To better understand the complexity of MS pathology and explore potential therapeutic strategies, various animal models have been developed (Dedoni et al., 2023). The most widely utilized model is experimental autoimmune encephalomyelitis (EAE), which replicates the autoimmune aspects of MS. Initially characterized in monkeys, EAE is induced by immunization with myelin-derived self-antigens such as myelin basic protein (MBP) or myelin proteolipid protein (PLP; Rivers et al., 1933). Presently, murine models are predominantly used to study both the relapsing–remitting and chronic progressive forms of EAE (Tuohy et al., 1989; Tompkins et al., 2002). Although EAE models have shed light on immune system activation and neuroinflammation, they provide a limited understanding of the CNS-intrinsic mechanisms underlying disease progression (Kamma et al., 2022). Other models, like toxin-induced demyelination using cuprizone or lysolecithin, enable investigation of demyelination and remyelination processes (Bénardais et al., 2013; Praet et al., 2014). Nevertheless, some studies using MS animal models have yielded disappointing clinical trial results, likely due to differences between rodent and human physiology (Mestas and Hughes, 2004). Currently, no effective treatments are available targeting repair and regeneration in pMS, highlighting the need for improved disease modeling approaches (Hollen et al., 2020).

Alongside animal disease models, *in vitro* human cellular methodologies have been developed to model and investigate MS pathology. Traditional 2D cell cultures offer a controlled environment for studying cellular interactions and disease mechanisms, overcoming some of the complexities associated with animal models, but they are limited in replicating the complexity of 3D tissue architecture (Dedoni et al., 2023). The development of 3D *in vitro* cultures, particularly human organoids, represents a significant advancement. Organoids can be generated from human pluripotent stem cells (hPSCs), including both embryonic stem cells (ESCs) and induced PSCs (iPSCs), as well as organ-specific adult stem cells. These cells self-organize into complex structures that mimic the cellular heterogeneity and organization of human organs (Gazerani, 2023). Patient- and disease-specific iPSCs can be used to generate specific cell types of interest allowing for the modeling and elucidation of specific cellular pathways implicated in disease. Human iPSC (hiPSC) technology is extensively employed to create human “disease-is-a-dish” models (Tang et al., 2022). The development of the first iPSC line, to our knowledge, from a patient with RRMS was in 2012, which marked a significant milestone (Song et al., 2012). Since then, advancements in generating MS-specific iPSCs have greatly enhanced the ability to study this complex neurological condition. Refined protocols now allow for the differentiation of iPSCs into neural progenitor cells (NPCs), neurons, oligodendrocytes, astrocytes, and microglia, offering promising avenues for understanding MS pathogenesis and accelerating the discovery of new therapies (Fortune et al., 2022b).

Brain organoids offer a novel platform for modeling MS pathology at a cellular level, with the potential to uncover disease mechanisms and develop new therapeutic strategies (Jusop et al., 2023). However, challenges remain, as brain organoids often exhibit cell immaturity, which limits their ability to accurately simulate intricate cellular interactions. Additionally, significant cell types like vascular cells, microglia, and immune cells, necessary for replicating neuroinflammatory mechanisms, are often lacking. Furthermore, incomplete maturation, underdeveloped neural circuits, and the inability to replicate aging processes further constrain their effectiveness in modeling neurodegenerative diseases. These limitations underscore the ongoing challenges in refining human brain organoid models for studying immune-mediated neurological diseases like MS (Kim and Chang, 2023).

This review will explore recent advances in brain organoid technologies and their application to MS research, aiming to enhance our understanding of disease mechanisms and guide the development of novel therapeutic approaches.

## 2 Current state and limitations of brain organoids

Traditional 2D cell culture models face significant limitations in accurately replicating *in vivo* cell environments, making them unreliable for disease modeling. In 2D cultures, cells adopt a flat and elongated morphology that contrasts with the natural 3D structure of tissues. This disparity is primarily due to reduced spatial organization and a lack of physiological conditions. The absence of crucial cell-to-cell and cell-to-extracellular matrix (ECM) interactions in 2D environments leads to altered gene and protein expression profiles, affecting the complex behavior of diseases (Sun et al., 2021). Additionally, these uniform conditions fail to replicate the inherent gradients of nutrients, oxygen, and waste essential for regulating cell behavior in living tissues.

Over the past few decades, a variety of 3D culture models have been developed, with spheroids and organoids becoming the most widely used. In a 3D culturing environment, cells can proliferate and interact with their surroundings, better mimicking the complex cellular interactions and tissue-specific architecture found in living organisms. This is typically achieved using multicellular aggregates, with or without a structural scaffold (Jensen and Teng, 2020). The shift from conventional 2D to 3D cell culture methodologies has accelerated in recent years, showing significant progress in generating spheroids, organoids, and assembloids. This advancement holds great promise for bridging the gap between 2D cell culture technologies and experiments with animals (Paiva et al., 2023).

### 2.1 Spheroids

Spheroids were initially developed in the 1970s and are simple clusters that can be created from different cell lines including primary cultures and stem cells. They possess the ability to spontaneously self-assemble into 3D aggregates that arrange into sphere-like formations. The generation of both single-cell and multicellular spheroid models relies on homotypic cell-to-cell adhesion in an environment that inhibits cell attachment to the culture surface (Sakalem et al., 2021). There are various methods available for creating spheroid cultures including the hanging drop and magnetic levitation technique, microfluidic systems, non-adhesive hydrogels and bioprinting. This 3D culture system finds key applications in drug testing and development, disease modeling and regenerative medicine (Białkowska et al., 2020; Griffin et al., 2022). However, due to the relative simplicity of spheroids, they lack polarity and the ability to self-organize into more complex tissue architectures, thus making this model less representative of accurate 3D *in vivo* conditions (Duval et al., 2017).

### 2.2 Brain organoids

3D brain organoids can exhibit complex architecture encompassing several neural and glial cell types, synaptic connections, and myelination. This 3D culture system mimics the intricacy of human neurodevelopment and brain diseases within a human setting, providing an unprecedented opportunity for *in vitro* human brain research. First described by Lancaster et al. in 2013 (Lancaster et al., 2013), the protocol outlines the natural self-organizing ability of stem cells into complex organ structures within a 3D ECM. By integrating specific growth factors, the protocol facilitates the neuroectodermal lineage differentiation of iPSCs. During 1 to 9 months, brain organoids mature into different brain areas and cell types, mimicking the development of the human brain *in vivo* (Lancaster and Knoblich, 2014). Since this initial discovery, many varying protocols have been established to mimic either specific brain areas (guided protocols) or the entire brain (unguided protocols). In the context of unguided differentiation, stem cells autonomously differentiate into organoids with distinct cell types and different brain regions, independent of external stimuli (Lancaster et al., 2013; Lancaster and Knoblich, 2014). In contrast, guided protocols entail the addition of extrinsic factors to imitate the morphogen gradient seen during the development of the embryonic brain, thereby conferring the organoid identities exclusive to certain regions (Eiraku et al., 2008; Kadoshima et al., 2013; Paşca et al., 2015). Moreover, the specific pattern development is also highly reliant on the strength and length of exposure to diffusible morphogens and the intricate interplay between various signaling pathways (Abdel Fattah et al., 2023). A diverse array of morphogen combinations from distinct signaling pathways has been employed to induce the formation of region-specific organoids, such as dorsal (Sebastian et al., 2023) and ventral forebrain (Zayat et al., 2023), hindbrain (Valiulahi et al., 2021), cerebellum (Atamian et al., 2024), spinal cord (Zhou et al., 2024) and choroid plexus (Pellegrini et al., 2020; Chew et al., 2023; Zhao and Haddad, 2024).

### 2.3 Advantages and limitations of brain organoids

The ability of brain organoids to self-organize during growth creates a 3D human brain environment with patient-derived genomes and evolutionarily conserved pathways, which are key advantages (Smirnova and Hartung, 2024). This 3D cell culture technology captures unique hallmarks of human brain development, including progenitor zone structures and neural migration and differentiation (Pamies, 2017). Alongside the existence of diverse mature brain cell types such as neuronal and glial cell varieties that replicate neuronal-glial communication and its connectivity, organoids show great promise for their application in disease modeling and precision medicine (Lancaster et al., 2017).

Over time, this methodology has become a new tool to model and investigate underlying mechanisms in neurodegenerative diseases such as pMS (Daviaud et al., 2023). Mutations can be precisely introduced into brain organoids via viral vectors or electroporation (Qian et al., 2019) to study the risk variants significance to disease in human tissue while pairing this approach with high-throughput screening techniques and *in vitro* single-cell profiling (Eichmüller and Knoblich, 2022). Moreover, brain organoids are proven advantageous in determining the neurotoxicity of medications and environmental contaminants. Conventional models such as 2D cell cultures are often unsuccessful in accurately predicting the effects of neurotoxins in humans because their uniform environment leads to altered cellular responses upon introduction of the neurotoxins (Bal-Price et al., 2010; Semple et al., 2013). In the context of precision medicine, brain organoids could potentially facilitate personalized therapy testing on patient-derived models, enabling patient-specific drug screening before clinical intervention, thus creating a new approach akin to “clinical trials in a dish” (Smirnova and Hartung, 2024). This approach could enable regular assessment of treatments on patient-derived models, helping to determine the most effective individualized course of action. Although organoid research is still in its early stages, it presents unprecedented possibilities for tailoring treatments and improving therapeutic outcomes beyond conventional methods (Yuan et al., 2023; Acharya et al., 2024).

However, it is essential to acknowledge the limitations of 3D brain organoids in studying and modeling diseases for the investigation of their underlying mechanisms. The capacity of this human experimental system to precisely mimic brain function and disease is limited by the lack of all cell types present in the human brain (Andrews and Kriegstein, 2022). Brain organoids predominantly comprise cells originating from the neuroectodermal lineage, constraining non-neuronal differentiation such as the mesodermal-derived microglia and vascular cells. This limits their significance and suitability as microglia play a crucial role in immune defense during disease and homeostasis (Ormel et al., 2018). The absence of vascularization impairs the efficient delivery of nutrients and oxygen to cells within the organoids, resulting in cellular stress, oxygen deprivation and necrosis (Kim and Chang, 2023). In contrast, the inclusion of endothelial cells (ECs) and vascular-like structures in brain organoids reduces cellular apoptosis and enhances neurogenesis (Andrews and Kriegstein, 2022). The organoids’ ability to expand is significantly restricted by the absence of the surrounding meninges and their associated vasculature. This deficiency results in a stochastic growth model driven by the presence of nutrients and the lack of complete spatial organization necessary for proper neural tissue patterning (Lancaster and Knoblich, 2014). Although these models closely resemble various essential aspects of early human brain development, they lack advanced brain activity due to the absence of sensory input and functional neuronal connectivity. Modeling age-dependent neurodegenerative diseases remains challenging because organoids do not naturally age or experience the same long-term environmental and genetic factors that contribute to these diseases *in vivo*. The ability to artificially induce aging *in vitro* could improve brain organoids’ capacity to exhibit phenotypes relevant to disease. The variability and reproducibility of organoid models present significant challenges due to differences in culture conditions, developmental trajectories, and the lack of standardized protocols, which can result in inconsistent outcomes. To overcome these issues, it is crucial to use multiple stem cell lines to minimize genetic variability, clearly differentiate between technical and biological replicates, and establish standardized reporting procedures. Additionally, adopting advanced technologies, such as high-resolution microscopy and single-cell sequencing, is recommended to enhance repeatability and transparency in the documentation of experimental conditions. Implementing these strategies can improve the robustness and consistency of organoid models, thereby advancing our understanding of human brain development and disease (Velasco et al., 2019; Hong et al., 2024; Sandoval et al., 2024). Despite these challenges, organoids remarkably resemble the *in vitro* development of the human brain and have shown widespread application across various fields of neuroscience research (Qian et al., 2019; Smirnova and Hartung, 2024).

### 2.4 Assembloids

Assembloids are generated by combining multiple spheroids or organoids derived from different types of cells or tissue areas. Human cortical spheroids have been assembled with human subpallium spheroids to potentially mimic more intricate elements of brain connectivity and development, using a co-culture technique. This has provided significant insights into the development of human cortical circuits (Birey et al., 2017). Other advanced techniques to generate assembloids include the fusion of organoids, referring to the physical combination of several organoid types to investigate inter-organ communication (Kanton and Paşca, 2022) and microfluidics. Brain region-specific organoids can be efficiently created by encasing hiPSCs within microcapsules using microfluidic electrospray methods. These organoids are then incorporated into a microfluidic chip, which includes an adjustable upper layer with a paired microhole array and a lower layer with a micropillar array. In these microholes, organoids fuse together and form intricate brain assemblies (Zhu et al., 2023). The unique advantage of these methods lies in their ability to elucidate how various tissue regions interact to exhibit novel biological characteristics. Assembloids hold great potential for simulating complex cell–cell interactions within and between tissues, helping to uncover the underlying pathophysiological mechanisms in disease and development (Kanton and Paşca, 2022). The assembly of brain and blood vessel organoids has shown significant promise for disease modeling; the inclusion of vascular-like structures enhances the organoid’s ability to simulate the nutrient and oxygen dynamics found *in vivo*. This advancement improves the organoid’s potential to replicate disease-relevant conditions, particularly for modeling complex neurological disorders (Dao et al., 2024).

## 3 Modelling MS *in vitro* with stem cell technology

The development of reliable *in vitro* or *in vivo* preclinical disease models is currently hampered by an incomplete understanding of the underlying mechanisms of MS. While the etiology of MS is not fully understood, it is believed to involve a combination of environmental factors, lifestyle, and genetic predispositions (Daviaud et al., 2023). The polygenic complexity of MS is underscored by genome-wide association studies (GWAS), which have identified 233 genetic variations frequently linked to the disease, collectively contributing to 39% of the genetic component of MS risk. This complexity complicates the development of exclusively genetic MS models (Barrie et al., 2024).

However, advances in human stem cell technologies have enabled the generation of patient-derived iPSCs, offering a novel model system to study this complex disease. MS iPSC lines consistently express pluripotency markers and have the capacity to differentiate into the three germ layers (Miquel-Serra et al., 2017; Lopez-Caraballo et al., 2020; Begentas et al., 2021; Fortune et al., 2022a; Lotila et al., 2022). Researchers have further differentiated these MS-derived cells into various cell types, including NPCs/neural stem cells (NSCs), neurons, OPCs, oligodendrocytes, astrocytes, brain microvascular ECs (BMECs), and brain organoids (Tables 1, 2).

**Table 1:** Different applications, advantages and disadvantages of 2D cultures (MS patient-derived iPSCs) to model and study MS pathology.

Type of MS	Number of lines generated	Age / Gender	Conclusion	Reference
SPMS	4	39 (F), 43 (F), 42 (F), 40 (F)	**iPSCs** express pluripotency markers differentiate into the three germ layers	Lopez-Caraballo et al. (2020)
RRMS	1	25 (M)		Begentas et al. (2021)
N/A	6	33 (F), 44 (M), 41 (M), 49 (F), 42 (F), 49 (F)		Miquel-Serra et al. (2017)
N/A	1	64 (F)		Lotila et al. (2022)
PPMS	1	65 (M)		Mehta et al. (2021)
PPMS	3	61 (M), 62 (F), 45 (F)	**NPCs and iNSCs** show signs of premature cellular senescence linked to inflammation and hypermetabolism	Nicaise et al. (2017)
PPMS	3	61 (M), 62 (F), 45 (F)		Nicaise et al. (2019)
RRMS (4), PPMS (4)	8	N/A		Mutukula et al. (2021)
pMS	5	25–63 (M/F)		Park et al. (2024) (bioRxiv)
PPMS (1), SPMS (3)	4	25–63 (M/F)		Ionescu et al. (2024) (bioRxiv)
RRMS	1	35 (F)	**Neurons** show typical morphology and functional competence	Song et al. (2012)
RRMS	1	32 (M)		Massa et al. (2016)
RRMS	4	15 (M), 17 (F), 21 (F), 31 (F)	**Astrocytes** pro-inflammatory and have an altered metabolism	Perriot et al. (2018)
RRMS (4), SPMS (2)	6	45 (F), 42 (F), 47 (F), 26 (F), 54 (F), 56 (F)		Ponath et al. (2018)
RRMS	3	61 (M), 35 (F), 56 (F)		Ghirotto et al. (2022)
RRMS (3), PPMS (2), SPMS (1)	6	F		Kerkering et al. (2023)
RRMS (4), SPMS (4), PPMS (4)	12	56 (F), 26 (F), 30 (F), 33 (M), 63 (F), 61 (F), 46 (F), 56 (F), 61 (F), 43 (F), 60 (F), 46 (M)		Clayton et al. (2024)
PPMS	5	62 (F), 50 (F), 56 (M), 61 (M)	**OPCs and Oligodendrocytes** inherent predisposition to adopt an immune-like phenotype show impaired myelination, altered gene expression, and dysfunctional cellular processes	Douvaras et al. (2014)
N/A	7	65 (F), 65 (F), 75 (M), 51 (F), 60 (M), 45 (F), 51 (F)		Smith et al. (2022)
RRMS	3	Disease duration at biopsy: 11 years, 16 months and 15 months		Mozafari et al. (2020)
N/A	3	N/A		Morales Pantoja et al. (2020)
RRMS	3	32 (F), 36 (M), 34 (F)		Starost et al. (2020)
PPMS	6	77 (F), 67 (F), 88 (F), 53 (F), 66 (M), 75 (M)		Plastini et al. (2022)
PPMS	2	56 (M), 62 (F)		García-León et al. (2020)
RRMS	4	15 (M), 17 (F), 21 (F), 31 (F)	**BMEC-like cells** display enhanced inflammatory phenotype exhibit impaired junctional integrity, compromised barrier properties, reduced efflux pump activity	Nishihara et al. (2020)
Advantages Simplified and Controlled Environment High Throughput Cost-Effective and Faster Disadvantages Limited Cellular Complexity Altered Physiological Conditions Inability to Model Complex Brain Development				

N/A, not available.

**Table 2:** Different applications, advantages and disadvantages of 3D cultures (MS patient-derived brain organoids) to model and study MS pathology.

Type of MS	Number of lines generated	Age / Gender	Conclusion	Reference
RRMS (2), PPMS (2), SPMS (3)	7	24 (M), 50 (F), 62 (F), 61 (M), 48 (M), 54 (F), 34 (F)	**Brain organoid** reduction in stem cell pool and proliferation capacity increase in number of neurons, decrease in number of oligodendrocytes	Daviaud et al. (2023)
RRMS	3	32 (F), 35 (M), 35 (F)	**Brain organoid with glia- enriched environment** transcriptomic profiles of comprised cell types akin to human adult brain microglia phenotype and microglia-astrocyte interaction similar to those in chronic active MS lesions	Fagiani et al. (2024)
Advantages Close Mimicry of *In Vivo* Conditions Human-Specific Disease Modeling Potential for Personalized Medicine Disadvantages Incomplete Cellular Composition Limited Maturation and Aging Technical Challenges and Variability				

### 3.1 MS iPSC-derived NPCs and iNSCs

NPCs are characterized by their tripotency, ability to self-renew and notable properties for neuroprotection and anti-inflammatory responses within areas of myelin damage after transplantation. However, NPCs derived from patients with pMS exhibit impaired stemness, fail to provide neuroprotection upon transplantation in cuprizone-lesioned mice, and show increased cellular senescence compared to controls (Nicaise et al., 2017, 2019; Mutukula et al., 2021; Daviaud et al., 2023). Recent advances in technology have enabled the investigation of NSCs in pMS through direct reprogramming, bypassing the pluripotent state when epigenetic markers of aging are erased, generating induced NSCs (iNSCs). These pMS iNSCs display senescent features associated with hypermetabolism, inflammatory signaling, increased cholesterol and long-chain fatty acid metabolism, accumulation of lipid droplets, and the secretion of a neurotoxic senescence-associated secretory phenotype (SASP; Ionescu et al., 2024). Single-cell transcriptomic and epigenetic profiling of these iNSC lines identified an IFN-responsive, highly senescent cell cluster in pMS iNSCs, also present in chronic lesions in the pMS brain (Park et al., 2024). Epigenetic dysregulation of IFN-response genes was found in both the original pMS fibroblasts and the iNSCs, suggesting that direct reprogramming preserves significant disease-related features. This method may serve as a valuable model for studying and understanding pMS mechanisms. Senescent and IFN-responsive NSCs may impact neighboring cells within pMS-affected brains and contribute to neurodegenerative processes.

### 3.2 MS iPSC-derived neurons

Primary neurons derived from people with RRMS were subjected to detailed electrophysiological analysis to assess the critical role of electrical signaling in neuronal function, particularly within the context of neurodegenerative conditions like MS. These differentiated neurons exhibited key characteristics of mature neurons, including stable resting membrane potentials and substantial action potentials in response to depolarization, as recorded using patch-clamp techniques. Morphologically, they displayed typical neuronal structures and expressed transcription factors and cytoskeletal markers indicative of neuronal differentiation (Song et al., 2012; Massa et al., 2016). These findings provide valuable insights into the functional integrity of neurons in MS and present a relevant approach to study and model disease-affected cell types.

### 3.3 MS iPSC-derived astrocytes

A few studies have generated and analyzed MS iPSCs-derived astrocytes with a focus on their phenotype, functionality and cell intrinsic inflammatory properties (Perriot et al., 2018; Ponath et al., 2018; Ghirotto et al., 2022; Kerkering et al., 2023; Clayton et al., 2024). These studies have identified differences in gene and protein expression linked to signaling pathways that govern neurodegeneration, mitochondrial dysfunction, inflammation, and cell death. These findings suggest intrinsic differences between MS iPSC-derived astrocytes and controls (Ghirotto et al., 2022). Metabolomic analysis further revealed abnormalities in amino acid synthesis and sphingolipid metabolism, indicating potential metabolic dysregulation in MS iPSC-derived astrocytes. Collectively, these findings suggest that MS-specific astrocytes assume a pro-inflammatory phenotype when exposed to cytokines expressed by microglia (Ghirotto et al., 2022; Kerkering et al., 2023). Recently, a study explored the properties of MS iPSC-derived astrocytes independent of inflammatory stimuli or the presence of other immune cells across all MS types. This research suggests that iPSC-derived astrocytes from patients across all MS subtypes acquire a pathological pro-inflammatory profile even in the absence of external inflammatory signals. This phenotype undermines their ability to support a neuroprotective environment, contributing to the progression of MS (Clayton et al., 2024).

### 3.4 MS iPSC-derived oligodendrocytes

Many protocols have been established to generate OPCs and oligodendrocytes from MS patients to investigate the mechanisms underlying remyelination failure in MS (Douvaras et al., 2014; García-León et al., 2020; Lopez-Caraballo et al., 2020; Morales Pantoja et al., 2020; Mozafari et al., 2020; Starost et al., 2020; Plastini et al., 2022; Smith et al., 2022; Clayton et al., 2024). In MS iPSC-derived oligodendrocytes and control subjects, equivalent outcomes were observed regarding OPC proliferation frequency, oligodendrocyte differentiation potential and myelin production in a 2D system (Starost et al., 2020; Clayton et al., 2024). However, when microglia-expressed cytokines (IFNγ and TNFα) were introduced to mimic an inflammatory environment, both control and MS-derived iPSC-OPCs exhibited impaired oligodendrocyte differentiation (Starost et al., 2020). Another study found that long-term exposure to low-dose IFNγ reduced oligodendrocyte formation in both MS-specific oligodendrocytes and controls (Morales Pantoja et al., 2020). This suggests that OPCs generated from MS iPSCs exhibit a differentiation block after exposure to inflammatory factors, similar to those derived from controls. However, proteome analysis of SPMS iPSC-derived OPCs revealed reduced production of specific proteins linked to cellular motility and cell-to-cell communication compared to the controls. Migration of MS-specific OPCs was found to be compromised, suggesting that the migration of OPCs in MS patients may be impaired (Lopez-Caraballo et al., 2020). Transcriptome analysis on PPMS-derived oligodendrocytes and controls identified numerous differentially expressed genes associated with inflammation, apoptosis, and cell adhesion. These findings highlight that while both MS- and control-derived oligodendrocyte lineage cells respond similarly to inflammatory factors, MS-derived cells have an inherent predisposition to adopt an immune-like phenotype and exhibit additional dysfunctions, such as impaired migration, which may contribute to MS-specific pathology (Plastini et al., 2022; Clayton et al., 2024).

### 3.5 MS iPSC-derived BMECs

Brain ECs constitute the BBB, a critical structure that regulates molecular trafficking and constrains the entry of immune cells into the CNS. ECs of the BBB undergo phenotypic alterations characterized by the upregulation of the cell adhesion and signaling molecules intracellular adhesion molecule-1 (ICAM-1), vascular cell adhesion molecule (VCAM)-1, and atypical chemokine receptor 1 (ACKR1) in people with MS (Zierfuss et al., 2024). This upregulation facilitates the increased infiltration of pathogenic immune cells into the CNS causing inflammatory damage to the BBB. The exact mechanisms underlying BBB failure are not fully understood, however, it is generally believed that neuroinflammation plays a significant role. A novel study generated MS iPSC-derived BMEC-like cells to mimic the BBB *in vitro* and to investigate the impact of BBB dysfunction in MS. BMEC-like cells derived from MS patients exhibited impaired junctional integrity, compromised barrier properties, reduced efflux pump activity, and an enhanced inflammatory phenotype, characterized by elevated expression of adhesion molecules and increased interactions with immune cells (Nishihara et al., 2020). These findings strongly suggest that MS patients possess intrinsic alterations in brain endothelium that contribute to BBB dysfunction, therefore iPSC-derived BMEC-like cells represent a valuable model for studying the underlying BBB dysfunction in MS. These models also hold the potential for identifying novel therapeutic targets aimed at stabilizing the BBB, which may be advantageous for treating both early and progressive stages of MS.

### 3.6 MS iPSC-derived brain organoids

Brain organoids were generated from iPSC lines derived from patients with RRMS, PPMS, and SPMS to better model and understand the pathological basis underlying the diverse clinical phenotypic expressions of MS. In the organoids generated from the progressive types of MS, a notable decrease in proliferation capacity was observed, accompanied by a reduction in the progenitor pool. This was also associated with increased neurogenesis and a decline in the number of oligodendrocytes (Daviaud et al., 2023). The absence of essential immune cells and vascularization poses limitations in accurately modeling the inflammatory demyelination and axonal degeneration associated with MS.

Another approach to modeling neuroinflammation in MS involves using MS and control NPC-derived forebrain-like organoids with forced expression of SOX10 to accelerate oligodendrocyte differentiation. Microglia are later transplanted into these 3D organoids. This setup results in the formation of neurons, astrocytes, oligodendrocytes, and microglia, with transcriptomic profiles similar to those of the adult brain. When exposed to cerebrospinal fluid (CSF) from MS patients, the microglia exhibit phenotypes resembling those found in chronic active MS lesions and show increased intercellular interactions with astrocytes (Fagiani et al., 2024). Despite not being strictly a brain organoid model, this relatively novel technology provides a scalable platform for drug screening, which can facilitate the discovery of novel therapeutics targeting the mechanisms underlying chronic inflammation and neurodegeneration in MS.

## 4 Emerging techniques to improve brain organoids for MS research

**Figure 2:**
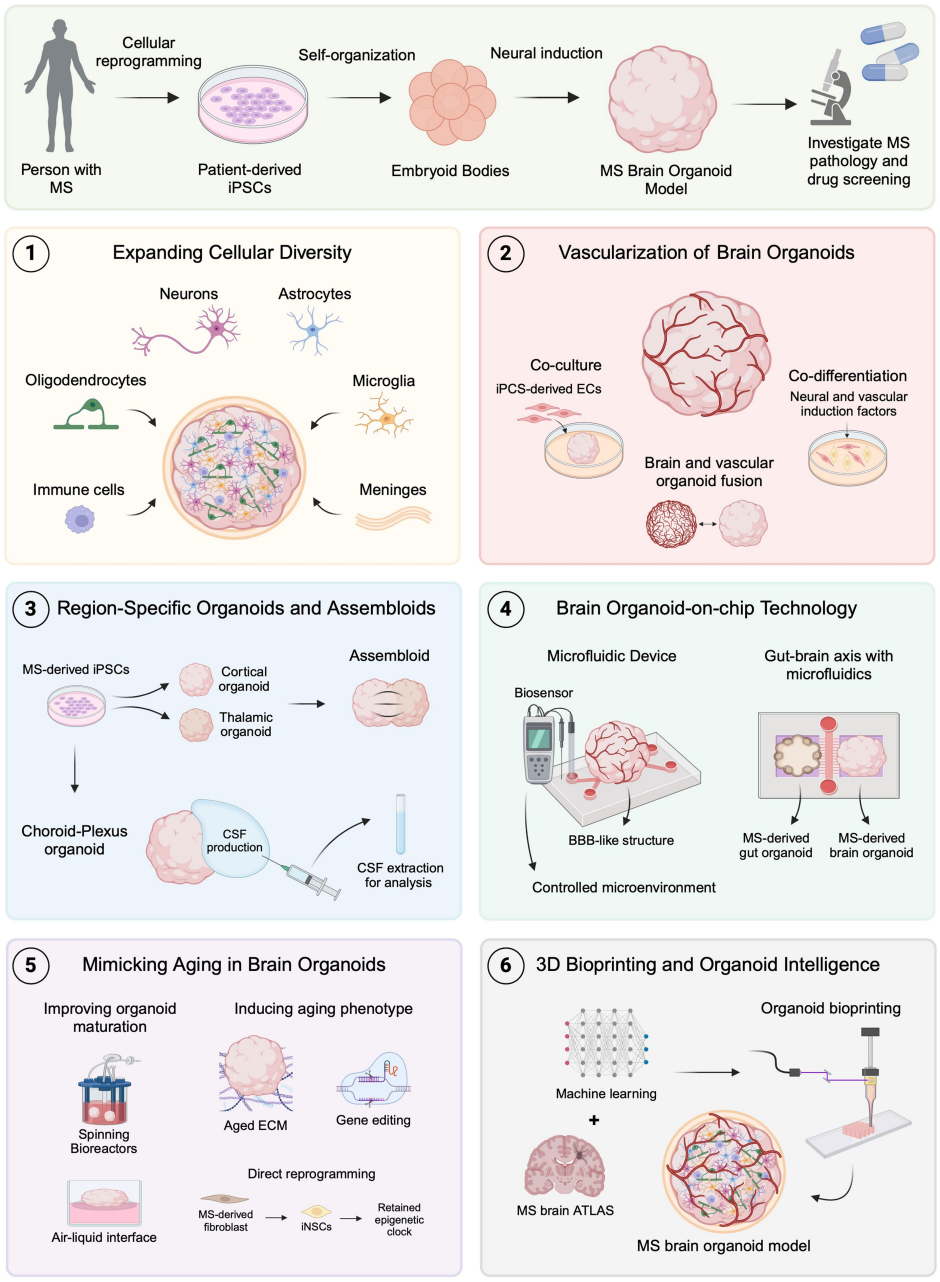
Emerging brain organoid techniques for modeling MS. MS brain organoids generated from patient-derived iPSCs offer a promising platform for studying MS pathology and drug development. (1) Expanding cellular diversity by incorporating oligodendrocytes, microglia, immune cells, and meningeal cells allows for more accurate modeling of MS-specific pathologies. (2) Advances in vascularization, including co-culture, co-differentiation, and organoid fusion, facilitate the formation of BBB-like structures to investigate MS-related BBB dysfunctions. (3) Region-specific organoids, such as choroid plexus, and assembloids, provide new avenues for studying CSF dynamics and neural circuit impairments in MS. (4) Organoid-on-chip technology using microfluidics creates controlled environments and can be used to replicate neuroinflammatory mechanisms and gut-brain axis interactions relevant to MS. (5) Techniques to mimic aging in organoids, including improved maturation protocols, show promise for studying age-related MS progression. (6) The combination of 3D bioprinting and machine learning with brain atlas data could lead to even more precise MS models, advancing the scope of *in vitro* disease modeling and therapeutic development. Created with BioRender.com.

Brain organoids have emerged as a valuable tool for disease modeling, offering a potential solution to the limitations of animal models in clinical translation. However, these 3D *in vitro* systems still face significant challenges, particularly in replicating the complex tissue microenvironment of the human brain. Recently, innovative bioengineering techniques have been developed to address these limitations, providing promising advancements in brain organoid models (Jeong et al., 2023). Additionally, these techniques introduce new approaches for modeling MS *in vitro*, potentially becoming valuable tools for studying pathological mechanisms and drug development (Figure 2).

### 4.1 Expanding cellular diversity in brain organoids

Most brain organoid models predominantly consist of NPCs, mature neurons, and astrocytes, yet they generally lack crucial cells such as oligodendrocytes, non-neuronal microglia, and peripheral immune cells (Del Dosso et al., 2020). This deficiency compromises the fidelity of brain organoids as accurate representations of the human brain. Incorporating these cell types is essential for developing accurate models of MS since infiltration of peripheral immune cells and oligodendrocyte death leading to demyelination are critical components of MS pathology (Compston and Coles, 2008). Additionally, in pMS, CNS-specific inflammatory mechanisms are believed to drive neurodegeneration (Lassmann et al., 2012), which underscores the significance of integrating microglia, the brain-resident macrophages, into brain organoids for modeling pMS and to better understand mechanisms driving disease progression.

Incorporating oligodendrocytes into brain organoids has been challenging due to the requirement for specific differentiation factors. However, protocols have been developed to promote oligodendrocyte maturation and myelin formation using targeted growth factors and hormones in human iPSC-derived neural models. For example, tailored differentiation protocols have successfully induced the formation of compact myelin sheets (Marton et al., 2019) and enhanced the survival, proliferation, and differentiation of OPCs in embedding-free brain organoids (Al-mhanawi et al., 2023; Shaker et al., 2021). More recently, a SOX10-based approach has generated brain spheroids that contain a stable pool of OPCs and MBP-positive oligodendrocytes exhibiting gene expression profiles resembling those of primary human oligodendrocytes (Fagiani et al., 2024). These advancements enable more accurate *in vitro* brain models by facilitating the formation of myelin sheaths, which are crucial for studying demyelination and remyelination mechanisms in MS. Myelin-enriched brain organoid models derived from patient iPSCs have been used to study myelin-related disorders and successfully recapitulated key pathological features (James et al., 2021; Feng et al., 2023). This highlights the potential for employing similar methodologies to investigate myelin pathology in MS. Notably, a study generated MS iPSC-derived brain organoids containing oligodendrocytes that showed decreased differentiation and myelination (Daviaud et al., 2023). This model offers a valuable tool for future research on drug efficacy in promoting remyelination, a process known to be impaired in the progressive stages of MS. Despite its innovation as a 3D *in vitro* model for MS, it lacks other important components of MS pathology, such as neuroinflammation.

In MS, activated microglia release various pro-inflammatory cytokines. These cytokines can be beneficial in the early stages of the disease but become detrimental as they contribute to persistent inflammation and neurodegeneration as the disease progresses (Zrzavy et al., 2017; Yong, 2022; Distéfano-Gagné et al., 2023; Peruzzotti-Jametti et al., 2024). The causes of chronic microglial activity in MS are not yet fully understood, and incorporating microglia into brain organoid models of MS could offer new insights into their role in pMS. Microglia originate from the yolk sac and migrate to the mesoderm during development, differing from neural and glial cells that arise from the neuroectodermal lineage. This developmental distinction has impeded the generation of microglia within neuroectoderm-derived brain organoids (Mosser et al., 2017). Recent advances have successfully integrated microglia into brain organoids using various techniques, creating new opportunities to study their role in MS (Mrza et al., 2024). Primary human microglia can incorporate into five-week-old brain organoids, maintaining homeostatic functions such as synapse remodeling without altering the overall structure of the organoid (Popova et al., 2021). Additionally, iPSC-derived microglia have been co-cultured with brain organoids, demonstrating activation in response to neuronal injury, a process relevant to MS pathology (Abud et al., 2017). Methods to generate microglia within organoids have also been developed. For instance, mesodermal progenitor cells can be differentiated into microglia by optimizing neuroectodermal stimulants (Ormel et al., 2018), and co-culturing hiPSC-derived neural and macrophage progenitors has shown promise in this area (Xu et al., 2021). These advancements underscore the potential for using MS-derived microglia in brain organoids to model disease mechanisms within a brain-like environment, providing insights into chronic neuroinflammation in MS. Recent work has further explored the role of microglia during neuroinflammation in a cortical organoid model of Alzheimer’s disease (AD; Cakir et al., 2022). The overexpression of the myeloid-specific transcription factor PU.1 successfully induced the differentiation of microglia-like cells within the organoids. This approach offers a valuable tool for understanding the role of chronically activated microglia in persistent inflammation related to MS and for studying disease-associated genes expressed in microglia. Genetic mapping has revealed numerous MS susceptibility genes linked to microglia (De Jager, 2019).

Until now, no studies have incorporated immune cells other than microglia into brain organoids (Sabate-Soler et al., 2024). However, this has been reported in other organoids such as liver, gut, and lung by seeding cells together in a scaffold, co-culturing of organoid and immune cells and direct injection of immune cells into the lumen of organoids (Bogoslowski et al., 2023). These techniques show potential ways for integrating peripheral immune cells into brain organoids that could be used to study immune cell activation and infiltration into the MS brain. Even though the field is still far from accurately mimicking the complex immune microenvironment of the MS brain *in vitro*, recent advances in developing immunized brain organoids represent valuable, translatable platforms for MS research. Future studies could generate iPSC-derived lymphocytes, such as T-like-cells (Iriguchi et al., 2021), from MS patients or directly collect T-cells from blood samples (Fransson et al., 2009) and incorporate them into brain organoids to investigate the adaptive immune mechanisms in MS brain microenvironment, particularly in RRMS, where immune cell infiltration across the BBB is predominant. Additionally, these models could serve as valuable platforms for identifying potential therapeutic targets and developing more effective treatments for MS.

The meninges are membranes covering the CNS that act as protective barriers and have been shown to play a critical role in corticogenesis and brain development by releasing essential signaling factors that influence neuronal differentiation, migration, and the formation of cortical layers (Dasgupta and Jeong, 2019). In pMS, the meninges may facilitate immune cell infiltration and chronic inflammation is frequently observed in this region, which is thought to play a role in neuroinflammatory processes and disease progression, especially cortical grey matter pathology (Serafini et al., 2004; Choi et al., 2012; Magliozzi et al., 2023). The integration of the meningeal layers into brain organoids would not only enhance their development and maturation but could also serve as a potential *in vitro* model to study meningeal inflammation in pMS. A recent study successfully mimicked the meningeal environment by co-culturing human meningeal cells with cortical organoids (Jalilian and Shin, 2023). Within the first 24 h of co-culture, meningeal cells adhered to and encapsulated the organoids, closely mimicking the architecture of *in vivo* meningeal membranes. This integration significantly enhanced cortical layer organization and promoted functional maturation, resulting in a more physiologically relevant model of human brain development. This protocol provides a valuable platform for investigating the role of meningeal inflammation and neuroinflammatory processes associated with pMS.

### 4.2 Advances in brain organoid vascularization

The neurovascular system is crucial for brain development and function. Therefore, vascularizing brain organoids is important to enhance their fidelity and maturation, as, without an effective vascular system, oxygen and nutrient exchange become suboptimal, leading to cell death (Yu, 2020). Establishing a vascular network in brain organoids is particularly relevant for studying neurological diseases, including MS, where the integrity of the BBB is compromised, allowing immune cells to infiltrate the CNS and contribute to neuroinflammation and demyelination. *In vivo*, vascular system development relies on key transcription factors and signaling cues that facilitate the self-organization and vessel formation of ECs and perivascular cells such as pericytes (Risau, 1997). Replicating this system *in vitro* is challenging due to its complexity, but recent bioengineering approaches have shown promising results.

Co-culturing patient-specific iPSC-derived ECs with brain organoids during early developmental stages has been shown to generate robust vascularization, with ECs forming tubular structures that penetrate the organoids (Pham et al., 2018). This approach demonstrates the feasibility of creating patient-specific vascularized organoids, making them particularly relevant for MS research. For instance, vascularized organoids derived from MS patients could be developed to assess BBB properties, such as permeability, and to gain insights into disease mechanisms associated with BBB dysfunction. Another promising method is co-differentiation, where neural and vascular lineages are simultaneously induced, leading to integrated neurovascular networks. Vascular endothelial growth factor (VEGF) has been utilized to promote the formation of blood vessel-like structures in brain organoids while maintaining neuronal differentiation, resulting in BBB-like vascular structures (Ham et al., 2020). Subsequent treatment with Wnt7a has been shown to enhance the formation of pericyte-like cells around the vascular tubes, better mimicking the *in vivo* BBB structure.

Emerging genetic engineering approaches, such as orthogonal induction (OID), further allow for the simultaneous differentiation of hiPSC-derived ECs and neurons within a single culture system, resulting in patterned, vascularized cortical organoids (Skylar-Scott et al., 2022). In the future, OID could be further explored to induce the generation of a diverse array of cell types from the same cell line through different transcription factors, creating a 3D model that more accurately mimics the human brain. Specifically, applying OID with MS patient-derived iPSCs could significantly enhance *in vitro* disease modeling by facilitating the generation of diverse cell types relevant to MS pathology.

Finally, vascularized brain assembloids, created by fusing brain organoids with vascular organoids, offer a complete vascular structure containing ECs, smooth muscle cells, and pericytes, demonstrating functional BBB characteristics and promoting neural development (Ahn et al., 2021; Sun et al., 2022). These innovative methods can be further leveraged using MS patient-derived iPSCs to create vascularized organoids, providing valuable tools for studying MS-associated BBB dysfunction and disease mechanisms.

In 2020, a study generated a 3D neurovascular unit organoid comprising neurons, oligodendrocytes, astrocytes, microglia, human brain microvascular ECs, and pericytes to study the effects of neuroinflammation on BBB function (Nzou et al., 2020). Exogenous inflammatory cytokines were found to increase BBB permeability, replicating characteristics of BBB dysfunction associated with neuroinflammation in pathophysiological conditions. For MS research, this approach could be adapted to explore BBB dysfunction driven by neuroinflammation seen in MS by using patient-derived organoids. Additionally, exposing these robust brain organoid models to the CSF from MS patients, which contains elevated levels of inflammatory mediators (Deisenhammer et al., 2019), would allow for a direct evaluation of how disease-specific inflammatory factors impact BBB permeability and function, revealing new insights into MS pathology. Furthermore, such models could be crucial for high-throughput drug screening to identify compounds that restore BBB integrity, like previous studies done in cell-based BBB models for AD (Qosa et al., 2016; Elfakhri et al., 2019). By more accurately replicating the neurovascular environment observed in MS, these advancements will contribute to the development of refined *in vitro* models, thereby accelerating the preclinical evaluation and discovery of targeted therapeutic strategies. Emerging methods such as incorporating brain organoids into microfluidic devices and employing 3D bioprinting offer promising avenues for creating more robust vascularized organoids, which will be explored further in the following sections (Nwokoye and Abilez, 2024; Rizzuti et al., 2024).

### 4.3 Region-specific brain organoids and assembloids

Previous studies have demonstrated the potential for generating region-specific brain organoids through the application of defined extrinsic signals (Qian et al., 2018), in contrast to conventional cerebral organoids that represent broad brain structures (Lancaster et al., 2013). These refined induction protocols can be applied in MS research to model specific brain regions and elucidate region-specific disease mechanisms. Although immune cell infiltration through the disrupted BBB is reduced in pMS, other structures, such as the choroid plexus (ChP), may continue to contribute to peripheral immune cell infiltration and neuroinflammation at this disease stage (Lassmann et al., 2012). Utilizing region-specific brain organoids to study the ChP could provide valuable insights into its role in pMS pathology.

In 2020, a study described a method for generating ChP organoids from hPSCs, which demonstrated the secretion of CSF-like fluid in self-contained compartments (Pellegrini et al., 2020). These organoids exhibited transcriptomic and proteomic profiles closely resembling those of the *in vivo* ChP structure, highlighting their potential for studying ChP-related disease mechanisms in pMS by generating MS-derived ChP organoids. Additionally, a recent study developed techniques for extracting and analyzing fluid from hPSC-derived ChP organoids using either syringe or centrifugation methods (Chew et al., 2023). These approaches could be utilized to analyze cellular response mechanisms to CSF-like fluid from ChP organoids derived from pMS patients, potentially offering valuable insights into ChP-CSF dynamics and their role in MS pathology.

Recent progress in assembloid technology has enabled the fusion of organoids from distinct brain regions, offering enhanced models to investigate brain function and disease mechanisms (Marton and Pașca, 2020). This approach is particularly relevant for studying diseases like MS, where interregional communication and connectivity are often disrupted. For instance, by fusing cortical and thalamic organoids, researchers generated thalamocortical organoids to explore thalamocortical circuitry dysfunction in psychiatric disorders (Kim et al., 2024). Given that MS is characterized by altered connectivity between the thalamus and cortex, and that thalamic atrophy has been linked to cognitive impairment in MS patients (Tona et al., 2014), generating patient-derived thalamocortical assembloids could provide critical insights into how MS-related disruptions in this circuitry contribute to cognitive deficits.

Such assembloids, when coupled with microelectrode array (MEA) technology, could also facilitate detailed analyses of connectivity dysfunction in MS, as discussed later. Recent technical advancements, such as acoustofluidics and 3D bioprinting, have improved the precision of organoid fusion. Acoustofluidic methods allow for the dynamic alignment and fusion of organoids with minimal disruption (Ao et al., 2021), while 3D bioprinting platforms like Spatially Patterned Organoid Transfer (SPOT) enable precise organoid assembly without compromising their structure (Roth et al., 2023). These techniques could be leveraged to develop more reproducible thalamocortical assembloids, furthering our understanding of MS-related functional deficits and facilitating the discovery of targeted therapeutic strategies.

### 4.4 Organoid-on-chip technology

Microfluidic chips are micro-engineered systems that provide precise control over fluid flow through spatially and temporally defined microchannels, creating controlled microenvironments that closely mimic *in vivo* conditions (Kang, 2022). These devices typically consist of multiple compartments or chambers with integrated pumps and valves that can be easily manipulated, allowing for the integration of various functions within a single chip (Kim et al., 2012; Leung et al., 2022). The appearance of organ-on-chip platforms has allowed *in situ* monitoring of the extracellular microenvironment using multisensor systems integrated into automated microfluidic controls (Zhang Y. S. et al., 2017). Recently, the development of brain organoids-on-chips has significantly improved brain organoid models by enhancing nutrient and oxygen exchange, which helps reduce necrosis and supports better maturation (Song et al., 2022). Additionally, advances in biosensors and optical imaging have significantly improved the monitoring of biochemical properties and cellular organization in organoid-on-chip models. Integrating enzyme-based biosensors allows continuous tracking of glucose and lactate levels (Misun et al., 2016), while optical techniques such as 3D tissue clearing and high-resolution imaging enable structural analysis of thicker tissues (Chen et al., 2016; Salman et al., 2020). These innovations could facilitate real-time monitoring of disease-specific changes in MS brain organoid models.

Recent studies have highlighted the potential of microfluidic systems to create perfusable vascular networks within brain organoids (Tan et al., 2023). Additionally, these devices can replicate the physiological conditions of the BBB, including the shear stress exerted by blood flow on ECs (Chaulagain et al., 2023). This shear stress is essential for enhancing EC maturation and promoting the formation of vessels with BBB properties (DeStefano et al., 2017). As a result, microfluidic systems offer a more realistic *in vitro* representation of the human BBB compared to static culture environments. Brain organoids were co-cultured with human umbilical vein ECs (HUVECs) within a microfluidic device, resulting in the formation of perfusable vascular networks that significantly enhanced neuronal differentiation and organoid maturation (Isshiki et al., 2020). In 2022, neurovascular organoids were developed using 3D-printed microfluidic chips, allowing for precise spatial interactions between organoid and vascular cells, which are essential for the establishment of an *in vivo*-like vascular network (Salmon et al., 2022). Furthermore, multiple studies have utilized microfluidic devices to construct vascular systems that exhibit BBB properties and functionality, facilitating their use in drug permeability screenings (Griep et al., 2013; Wang et al., 2017; Papademetriou et al., 2018; Wevers et al., 2018). This could be applied to test potential drugs for MS, and their efficiency in crossing the BBB, especially for pMS, in which pathological mechanisms are CNS-exclusive (Absinta et al., 2020).

Microfluidic devices have significantly enhanced brain organoid models and their vascularization, providing more accurate and physiologically relevant platforms for studying neurological disorders (Amartumur et al., 2024). These systems have already been applied to investigate BBB dysfunction in neurodegenerative diseases such as AD and Parkinson’s disease (PD; Shin et al., 2019; Pediaditakis et al., 2021) and similar approaches could be used in MS research. Recent studies have demonstrated the potential of microfluidic systems to explore neuroinflammatory disease mechanisms. For example, a 3D BBB chip developed for PD research utilized a model composed of BMEC-like cells, pericytes, and astrocytes, separated by an ECM gel, allowing for a detailed investigation of how pro-inflammatory astrocytes disrupt vascular integrity (de Rus Jacquet et al., 2023). This model could be adapted for MS research to examine the role of disease-associated astrocytes and inflammation in BBB dysfunction. Similarly, a 3D microfluidic platform designed to study neuroinflammation in AD used a circular design with central and radial chambers to facilitate the migration of microglia toward areas enriched in AD-related cytokines, such as TNFα, IL-1, and IL-6 (Park et al., 2018). Adapted for MS, such a platform could help investigate how MS-related inflammatory signals influence microglia activation and neuroinflammation. These innovative microfluidic systems offer powerful tools for studying the complex interactions between glial cells, neurons, and the vascular environment.

Recent advances in microfluidic technology have facilitated the integration of brain organoids with peripheral organoid models, offering a more comprehensive approach to studying inter-organ interactions. This is particularly important in neurodegenerative diseases where systemic factors and peripheral organs play a role in disease progression. In MS, emerging evidence underscores the importance of the gut-brain axis in modulating disease pathology, particularly through microbial dysbiosis and immune system interactions (Sharifa et al., 2023). Microfluidic platforms have been used to create models that mimic the gut-brain axis, allowing researchers to investigate its role in health and disease (Moysidou and Owens, 2021). One study developed a modular microfluidic chip that co-cultures gut epithelial and brain ECs to form interconnected barriers (Kim et al., 2021). This setup allowed researchers to observe the transport of fluorescently labeled exosomes across the gut-brain barrier, providing insights into the communication pathways between the gut and brain. Another study introduced a two-compartment microfluidic device designed to co-culture enteric neurons and intestinal epithelial cells (de Hoyos-Vega et al., 2023). This device enables the examination of neuro-epithelial interactions, revealing how epithelial cells can influence neuronal projections and connectivity. These advanced microfluidic systems enable researchers to investigate how conditions in peripheral organs, such as gut dysbiosis, impact brain function and contribute to MS progression. Additionally, they offer the potential to uncover new insights into the systemic factors influencing MS and evaluate therapeutic strategies targeting the gut-brain axis. Future developments, such as gut-brain organoids-on-chips, hold promise to further enhance the relevance and accuracy of these models.

### 4.5 Modeling aging with brain organoids

Modeling aging in brain organoids is critical for developing more accurate models of age-related neurological diseases, particularly for pMS, where aging is the primary risk factor (Sanai et al., 2016). Traditional brain organoids often fail to fully capture the complexities of aging. However, recent advancements in organoid maturation techniques offer promising strategies to enhance the representativeness of these models.

Extending the culture period of brain organoids is a fundamental approach to improve their maturation, supporting the development of mature neuronal populations and complex neural networks that more closely resemble the aged human brain. Bioreactors are crucial for sustaining long-term cultures by optimizing nutrient supply and oxygenation. Spinning bioreactors provide a low-shear environment that ensures uniform nutrient distribution through continuous motion, improving organoid viability and cortical structure organization (Lancaster et al., 2013). Miniaturized multi-well spinning bioreactors have also been developed, enhancing reproducibility and increasing the throughput of organoid production (Qian et al., 2016, 2018). Moreover, innovations such as 3D-printed bioreactor molds offer the ability to precisely shape and structure tissues or organoids during their growth, enhancing their reproducibility (Priyadarshini et al., 2020). The air-liquid-interface culture system is another technique used to improve long-term culture. This method has been shown to enhance organoid maturation, cell survival, and structural organization by improving nutrient and oxygen exchange (Giandomenico et al., 2019). These advanced techniques offer promising methods for generating organoids that more accurately represent the aging brain. Furthermore, MEAs can be used to assess neural networks within organoids, providing a valuable tool for monitoring their maturation and evaluating age-related changes over time (Obien et al., 2015). These advancements enhance our ability to model aging in brain organoids, providing more accurate representations of age-related changes and disease mechanisms. This progress is especially significant for pMS research, as it could provide deeper insights into age-driven pathological changes and aid in the identification of novel therapeutic strategies.

In addition to maturation techniques, it is important to induce specific aging phenotypes in brain organoids to create models that more closely resemble the biological processes associated with aging. Modifying the ECM by incorporating aged ECM components can replicate the age-related changes in cell adhesion, signaling, and tissue stiffness, which are key factors in aging (Ozcebe et al., 2021). Cellular manipulation techniques, such as telomerase activity modulation and CRISPR-Cas9 gene editing, or the use of ionizing radiation, also play a critical role in mimicking the biological features of aging, such as mitochondrial dysfunction, oxidative stress, and DNA damage (Vera et al., 2016; Acun and Zorlutuna, 2019; Oyefeso et al., 2023). Another promising approach involves using aged iPSCs, which retain intrinsic metabolic shifts and mitochondrial dysfunction characteristic of aging (Zhang C. et al., 2017). Direct reprogramming techniques that maintain the epigenetic clock offer another method, as they preserve aging signatures and avoid the rejuvenation typically seen in iPSC reprogramming (Tang et al., 2017). These approaches offer versatile methodologies for generating aging brain organoid models that could be used to study age-related neurodegenerative diseases, including pMS. For instance, recent studies have utilized direct reprogramming to generate iNSCs from MS patients, successfully preserving the aging phenotype characteristic of *in vivo* pMS NSCs, as previously discussed (Ionescu et al., 2024; Park et al., 2024). This approach holds promise for advancing 3D *in vitro* models of MS, potentially leading to more accurate representations of pMS pathology.

Microfluidics have advanced the study of age-related mechanisms by providing precise control over the cellular microenvironment and allowing for customizable experimental conditions. For instance, a recent study developed a human brain organoid microphysiological analysis platform (MAP) to investigate immune-driven brain aging (Ao et al., 2022). This platform incorporates dynamic rocking flow to introduce primary monocytes from both young and aged donors into mature organoids, effectively modeling neuroimmune interactions. The study found that aged monocytes increased their infiltration into the organoids and enhanced the expression of aging-related markers, suggesting that these cells may contribute to brain aging processes. This platform demonstrates the potential of microfluidic devices for studying aging mechanisms in the brain. Introducing monocytes derived from pMS patients into this system could offer novel insights into their contribution to disease progression, particularly in immune-driven neurodegeneration, by assessing disease-specific alterations within brain organoid models in the MAP. This approach is especially relevant given that non-classical monocytes, including the CD14^+^/CD16^++^ subset, have been implicated in the initiation and progression of MS (Arneth, 2024). Leveraging this model could provide a valuable platform for unraveling the complex dynamics of these immune cells in MS pathology, potentially guiding the development of targeted therapies.

### 4.6 MEAs to study brain organoid networks

MEAs are sophisticated tools for recording and monitoring the electrophysiological activity of neurons *in vitro*, offering precise insights into neural dynamics, synaptic transmission, and network connectivity (Obien et al., 2015). These platforms consist of multiple electrodes that detect extracellular ionic currents, enabling the capture of action potentials and providing high-resolution data on neuronal activity and interactions within neural circuits. While extensively used in traditional neuroscience research, MEAs have recently been applied to brain organoids, offering a minimally invasive method to evaluate neural network maturation and functionality over time with high temporal and spatial resolution (Trujillo et al., 2019; Sharf et al., 2022). This capability of long-term monitoring of neural networks is particularly valuable for studying network alterations in 3D models of neurological diseases, facilitating the analysis of disease-related network dysfunctions and potential therapeutic targets. For instance, MEA has been used to assess neuronal networks in a midbrain organoid model of PD, which revealed increased neuronal firing frequency, consistent with pathophysiological characteristics of PD (Wulansari et al., 2021). Altered network connectivity has already been described in MS (Schoonheim et al., 2022), and applying MEAs to MS-specific brain organoids could provide valuable insights into the precise neural connectivity disruptions associated with the disease.

Traditional 2D MEAs, which utilize a planar array of electrodes, have been instrumental in capturing early network formation, such as increasing electrical activity and oscillatory waves during cortical organoid maturation (Trujillo et al., 2019). High-density MEAs (HD-MEAs), with their dense grids of hundreds to thousands of electrodes, enhance spatial resolution, enabling detailed analyses of neural connectivity. For instance, HD-MEAs have revealed networks with numerous weak connections and fewer strong, functional links in hiPSC-derived brain organoids (Sharf et al., 2022) and provided extensive electrophysiological data from sliced organoids, allowing precise tracking of neurons and functional connectivity (Schröter et al., 2022). Despite their advancements, 2D MEAs are limited to surface-level recordings, restricting access to the complex internal regions of 3D organoids. To overcome this limitation, innovative 3D MEAs have been developed to capture neural activity across all tissue layers, offering a comprehensive view of neural network functionality. Technologies such as shell-shaped MEAs enable long-term monitoring of activity across multiple organoid regions (Huang et al., 2022), while flexible 3D MEAs integrate within the organoid matrix for sustained, non-invasive recordings of internal neural dynamics (Soscia et al., 2020). Further advancements, including mesh microelectrode arrays and stretchable nanoelectronics, seamlessly adapt to growing organoid tissue, providing high-resolution data without disrupting development (McDonald et al., 2023; Le Floch et al., 2022; Stoppini et al., 2024). Advanced 3D flexible and stretchable MEA technologies hold significant potential for future research into neural network connectivity within brain organoid models of MS. By adapting to the mechanical properties of brain organoids, these devices enable continuous, high-resolution recordings throughout organoid development, allowing researchers to monitor and analyze neural network changes from early stages through advanced disease stages. This capability can greatly enhance our understanding of how aging and disease progression impact neural connectivity in MS. Additionally, using microglia-enriched patient-derived organoid models with MEA techniques could provide deeper insights into the impact of smoldering inflammation on network integrity in pMS. Furthermore, applying organoid intelligence techniques to replicate cognitive processes in brain organoids, as recently suggested (Smirnova et al., 2023), could reveal how specific network alterations contribute to cognitive deficits typically seen in MS patients.

### 4.7 3D bioprinting and organoid intelligence

3D bioprinting has emerged as a transformative technology for fabricating tissues that closely replicate *in vivo* architecture (Zhang et al., 2019). This technique employs bioinks, composed of various cell types and biomaterials, to create precise, layer-by-layer constructs that mimic natural tissues, presenting extensive potential for tissue and organ engineering (Gungor-Ozkerim et al., 2018). Beyond its traditional applications in organ transplantation and tissue repair, 3D bioprinting has shown significant promise in generating multiple organoids with high reproducibility, enabling the development of accurate 3D *in vitro* models for studying a wide range of diseases (Ren et al., 2021).

Several studies have highlighted the potential of 3D bioprinting for creating complex neural tissues. For instance, a pioneering study used a polysaccharide-based bioink to bioprint hiPSCs, achieving successful encapsulation, *in situ* expansion, and differentiation into functional neural tissues with synaptic activity and migratory behavior (Gu et al., 2017). Replicating self-organized patterns of cortical development in a controlled and spatially organized manner *in vitro* remains challenging. Although 3D bioprinting offers a customizable approach, existing materials often fail to accurately mimic the ECM of brain tissue. To address this, one study developed a lipid-bilayer-supported 3D printing method using Matrigel, a soft and biocompatible ECM (Zhou et al., 2020). This method enabled precise pre-patterning of human NSCs and astrocytes, resulting in high cell viability, effective differentiation, and functional neural networks, improving brain tissue modeling. Recently, 3D bioprinting approaches have been developed to address current limitations on brain organoids vascularization. By applying OID to pre-programmed iPSCs, researchers were able to create vascularized 3D neural tissues with distinct, spatially organized regions comprising NSCs, ECs, and neurons using multimaterial 3D bioprinting (Skylar-Scott et al., 2022). In a different approach, a recent study developed a 3D bioprinted microchanneled scaffold made of gelatin methacrylate, a biomaterial with ECM properties, for controlling cortical organoid development and patterning (Cadena et al., 2024). This scaffold supported the long-term culture of cortical brain organoids and enabled HUVECs to infiltrate the organoids, promoting their vascularization. These novel systems enable high-throughput and reproducible production of vascularized organoids, creating more physiologically relevant models for studying brain development and neurological disorders. Interestingly, a study developed an advanced 3D bioprinting technique using patient-derived iPSCs to model AD (Benwood et al., 2023). The constructs showed strong cell viability and functionality, highlighting the potential of this technique for creating accurate patient-specific neural models. This approach may also be applied to advancing patient-specific 3D *in vitro* models of other neurological conditions, including MS.

Organoid intelligence represents a groundbreaking integration of advanced organoid technology with artificial intelligence (AI), particularly machine learning (ML). This synergy facilitates comprehensive analysis of organoid behaviors, cellular interactions, and dynamic responses to stimuli, enabling the development of predictive models, precise disease simulations, and personalized medicine approaches (Shi et al., 2024). Recent advancements in AI and ML, combined with detailed human brain atlases, offer significant potential for enhancing 3D bioprinting of brain organoids (Lee, 2023; Chen et al., 2024). Brain atlases provide intricate maps of brain architecture and cellular organization, which ML algorithms can utilize to guide the spatial arrangement and differentiation of cells in bioprinted organoids. This integration enables the accurate modeling of complex brain structures, including disease-specific features. For instance, detailed molecular maps of lesion stages in pMS (Frisch et al., 2020) could be used to refine ML algorithms and create advanced 3D *in vitro* models for studying disease progression and optimizing drug screening. Additionally, recently described aging brain atlases (Yang et al., 2024) can be employed to replicate mature brain patterns and organization, which could be particularly valuable for studying pMS and other age-related neurodegenerative diseases. Collectively, these advancements underscore the transformative potential of organoid intelligence in significantly enhancing research and therapeutic approaches in neurological disorders. By leveraging sophisticated 3D brain organoid models, researchers can achieve more accurate representations of both healthy and diseased brain tissue, advancing our understanding and treatment of complex neurological conditions.

## 5 Conclusion and future perspectives

Brain organoids represent a significant advancement in 3D *in vitro* modeling, offering a sophisticated platform for exploring human brain development and pathology, with notable potential for advancing research into neurological disorders such as MS. Compared to traditional *in vitro* and animal models, organoids provide a more accurate replication of human brain architecture, including its complex cellular diversity, structural organization, and neurovascular system, making them particularly relevant for MS research. While the application of brain organoids to model MS is still emerging, the integration of advanced techniques, such as long-term cultures, 3D bioprinting, and microfluidics, holds promise for creating models that more closely mimic the complex pathology of MS. Additionally, the use of high-resolution tools, including MEAs and AI-driven algorithms, could greatly enhance our ability to investigate neural networks and disease-specific mechanisms. These advancements are crucial for deepening our understanding of MS pathophysiology, particularly in its progressive forms where current therapeutic options are limited. By simulating disease conditions more accurately, brain organoids facilitate the identification of potential therapeutic targets and improve drug screening processes, offering new avenues for developing effective treatments. Looking to the future, brain organoid technology holds significant promise for bridging the gap between fundamental research and clinical application. Patient-derived organoids could facilitate personalized medicine, offering tailored therapeutic strategies and advancing regenerative approaches for CNS repair. As brain organoid technology continues to evolve, it promises to significantly impact both MS research and clinical practice. Although still in its early stages, the ongoing advancements in this field point to a future where our understanding and treatment of MS could be fundamentally transformed, leading to groundbreaking discoveries and significantly improved patient outcomes.
